# Mild cognitive impairment predicts the onset of Sarcopenia: a longitudinal analysis from the English Longitudinal Study on Ageing

**DOI:** 10.1007/s40520-024-02781-z

**Published:** 2024-06-10

**Authors:** Francesco Saverio Ragusa, Nicola Veronese, Laura Vernuccio, Ligia J Dominguez, Lee Smith, Francesco Bolzetta, Ai Koyanagi, Roberto Monastero, Mario Barbagallo

**Affiliations:** 1 Geriatric Unit, Department of Internal Medicine and Geriatrics, University of Palermo, Via del Vespro, 141, 90127,, Palermo, Italy; 2 Faculty of Medicine and Surgery, Kore University of Enna, Enna, Italy; 3 Centre for Health, Performance, and Wellbeing, Anglia Ruskin University, Cambridge, UK; 4 Azienda Unita Locale Socio Sanitaria 3 Serenissima, Department of Medicine, Geriatrics Section, Dolo-Mirano, Italy; 5 Research and Development Unit, Parc Sanitari Sant Joan de Déu, Universitat de Barcelona, Fundació Sant Joan de Déu, Barcelona, 08830 Spain; 6 Department of Biomedicine, Neuroscience, and Advanced Diagnostic (BIND), University of Palermo, Palermo, Italy

**Keywords:** Cognitive impairment, ELSA, Sarcopenia, Older people, Longitudinal

## Abstract

**Background:**

Mild cognitive impairment (MCI) and sarcopenia are two common conditions in older people. It is not widely known if MCI could predict the onset of sarcopenia. Therefore, we aimed to investigate whether MCI could predict the occurrence of sarcopenia in a population of older adults.

**Methods:**

In the ELSA (English Longitudinal Study on Ageing), MCI was defined as the absence of dementia, preserved functional capacity and low performance in three objective cognitive tests. Sarcopenia was diagnosed as having low handgrip strength and low skeletal muscle mass index during follow-up. The longitudinal association between MCI at the baseline and incident sarcopenia was assessed using a multivariable logistic regression model, reporting the data as adjusted odds ratios (OR) and 95% confidence intervals (95%CI).

**Results:**

3,106 participants (mean age of 63.1 years; 55.3% males) were included. People with MCI reported significantly lower mean handgrip strength values and Skeletal Mass Index (SMI), as well as a higher prevalence of obesity at baseline. At baseline, 729 people had MCI and during the ten years follow-up period, 12.1% of the initial population included had sarcopenia. On multivariate analysis, adjusted for 18 potential confounders, the presence of MCI (OR = 1.236; 95%CI: 1.090–1.596, *p* = 0.01) significantly predicted the onset of sarcopenia during follow-up.

**Conclusion:**

The presence of MCI at baseline was associated with a higher incidence of sarcopenia at ten-years follow-up, demonstrating a likely role of MCI as a predictor of the onset of sarcopenia in older people.

## Introduction

Aging is a process that occurs naturally, inevitably, and involves changes in physical, mental, and functional capabilities, signalling a decline in the body’s ability to regenerate and repair itself [1]. It is well known that among older people keeping a balance of physical and psychic factors is fundamental to preserve homeostasis. Indeed, epidemiological studies show that 11% of the world’s population is over 60 years and this is projected to increase to 22% of the population by 2050 [2]. Currently, the age composition of the global population is undergoing a transformation as fertility rates decline and life spans increase [3].

Mild cognitive impairment (MCI) is a clinical construct based on subjective cognitive decline, objective cognitive impairment, and relative preservation of activities of daily living [4, 5]. MCI rates range from 3% to as high as 42% in population studies, and from 6 to 85% in clinical settings [6, 7]. The conversion rate to dementia is approximately 10% per year, which increases to 80–90% after approximately six years [8]. Clinical criteria for MCI commonly include impaired cognitive performance, preserved basic activities with minimal impairment in complex instrumental activities, self and/or informant report of cognitive decline, and absence of dementia [9]. Accordingly, MCI is to be considered a transitional phase between physiological ageing and dementia [10]. 

Sarcopenia is defined as age-related muscle loss, affecting a combination of appendicular muscle mass, muscle strength, and/or physical performance measures [11]. It is widespread among older adults, and the overall prevalence is approximately 10% in both men and women over 60 years [12]. Reduction in physical capacity and functional decline that could be caused by sarcopenia, can lead to a higher levels of dependency and disability and therefore negatively influence levels of health-related quality of life [13]. 

Some studies showed that MCI could be associated with a higher incidence of sarcopenia. A study in China of 5,715 participants demonstrated how incidence of MCI was higher in sarcopenic groups compared to non-sarcopenic groups, with a significantly statistical difference [14], but this study did not consider some potential important confounders, such as physical activity level. Another study in Mexico of 496 older adults observed a significant longitudinal association between sarcopenia, MCI and poor cognitive function among older patients. However, this study included a small sample size, with a clear predominance (65%) of female participants [15].

Previous literature has focused on the identification of MCI among individuals with sarcopenia and has not investigated whether MCI is associated with incident sarcopenia. Therefore, the aim of the present study was to investigate whether the presence of MCI at baseline predicts the occurrence of sarcopenia in older people at 10-years of follow-up, using data from the English Longitudinal Study on Ageing (ELSA).

## Methods

### Study population

This study is based on data from several waves (from Wave 2 to 7) of the ELSA, a prospective and nationally representative cohort of older community-dwelling participants living in England [16]. Wave 2 (baseline survey) was conducted in 2004–2005; the other waves were conducted every two years, until Wave 7 occurring between 2014 and 2015. The ELSA study was approved by the London Multicentre Research Ethics Committee (MREC/01/2/91). Written informed consent was obtained from all participants.

### Mild cognitive impairment: independent variable

Mild cognitive impairment (MCI) is defined as the presence of cognitive impairment and the absence of any functional impairment [9]. As suggested by Vancampfort et al. [17] we defined MCI as the absence of dementia, low cognitive performance on three objective cognitive tests (see below), and preserved functional capacity [18]. Participants who filled all the criteria were classified as having MCI.

Dementia was defined using the self-reported medical diagnosis of either Alzheimer’s disease or dementia [19]. Participants were considered with medical diagnosis of dementia if answered yes to the following question: “Has a doctor ever told you that you have (or had) dementia and/or Alzheimer’s?“.

In the ELSA study, three different cognitive tests were used to determine objective cognitive performance: 10-word list delayed recall [20], verbal fluency [21], and orientation for time [22]. Cognitive scores from a 10-word list recall test and verbal fluency were standardized into a z-score based on mean and standard deviation (SD) from the final sample. Objective cognitive impairment was considered present when observed scores were lower than 1 SD below the average in one or more cognitive tests [18]. Briefly:

- The 10-word list recall test consists of two tasks (immediate and delayed) and is part of the Consortium to Establish a Registry for Alzheimer’s Disease [20]. During the test, participants were asked to recall a list of 10 unrelated nouns in each task. The delayed task was performed about 20 min after the immediate recall task. The performance at both tasks is related to learning and episodic memory. We used the sum of both tasks to generate a standardized z-score. This test has good sensitivity and specificity for detecting MCI [23].
- Orientation for time consists of four questions about the current date, i.e., day of the month, day of the week, current month, current year. Cognitive impairment was considered when participants failed to identify either year or month correctly [18].
- Verbal fluency test: participants produced a list of all the animals he or she could remember in 1 min. One point was given to each uniquely named animal. This test involves verbal fluency and has good sensitivity and specificity to determine MCI [24].

Finally, functional capacity was assessed using self-reported difficulties with basic activities of daily living (ADL) in the last month [25]. If participants answered *no* to one of the following questions, functional capacity was considered preserved [17, 18]: “Have you received help for getting dressed?” and “Have you received help for eating (e.g. cutting up your food)?“.

### Incident sarcopenia: outcome

In the ELSA study, the evaluation of body composition was not carried out with gold standard measures, such as DXA (Dual-Energy X-ray Absorptiometry). Therefore, we used a surrogate measure of low fat-free mass for the estimation of body composition, defined as having a low SMI. SMM (skeletal muscle mass) was calculated based on the equation proposed by Lee and colleagues [26], i.e.: ASM = 0.244*weight + 7.8*height + 6.6*sex–0.098*age + race–3.3 (where female = 0 and male = 1; race = 0 (White and Hispanic), race = 1.9 (Black), and race = − 1.6 (Asian)). [9] Next, SMM was divided by body mass index (BMI) based on weight and height measured by a trained nurse, to create the SMI [27]. Low SMM was defined as the lowest quartile of the SMI based on sex-stratified values [28]. The equation proposed by Lee has been previously used in the ELSA study ( 29, 31) and validated against gold standard methods, such as DXA [32, 33]. 

The identification of sarcopenia was completed using, as indicator of low muscle strength, the presence of low handgrip strength defined as < 27 kg for men and < 16 kg for women using the average value of three handgrip measurements of the dominant hand [34]. Grip strength in kilograms was measured by using a Smedley dynamometer (TTM; Tokyo, Japan), with the upper arm being held against the trunk and the elbow in a 90-degree flexion [16]. 

### Other factors

The selection of other factors potentially associated with the relationship between MCI and sarcopenia was based on previous literature [35] and included the following: age; sex; years of education (considered as continuous variable); ethnicity (whites vs. non-whites); marital status (married vs. other status); smoking status (ever vs. never); and physical activity level (high vs. moderate/low/sedentary). The level of physical activity was assessed using three questions to assess vigorous, moderate, or mild activity in the previous twelve months. To assist in answering the questions, prompt cards with examples of activities categorized by intensity were used [36]; the presence of depressive symptoms was assessed using the Center for Epidemiologic Studies Depression Scale (CES-D) [37]; medical conditions were recorded based on self-reported information; and the presence of obesity was defined as having a BMI *≥* 30 Kg/m^2^ [37]. All these factors were assessed at the baseline.

### Statistical analysis

Continuous variables were described as mean and standard deviation (SD). Categorical variables were analysed as counts and percentages. Study participants classified by the presence or not of MCI at the baseline, were compared using Chi-squared or Fisher exact tests, for categorical variables and t-test, for continuous variables.

The longitudinal association between MCI at the baseline and incident sarcopenia was assessed using a multivariable logistic regression model, reporting the data as adjusted odds ratios (OR) and 95% confidence intervals (95%CI). The factors included were significantly different between MCI and no MCI at the baseline or they were associated with incident sarcopenia, at a p-value < 0.10. The collinearity among covariates was assessed using the variance inflation factor (VIF), using a cut-off of two as reason for exclusion, but no factor was excluded for this reason [39]. 

All statistical tests were two-tailed, and a p-value < 0.05 was considered statistically significant. All analyses were performed using SPSS 26.0 version software.

## Results

**Fig. 1:**
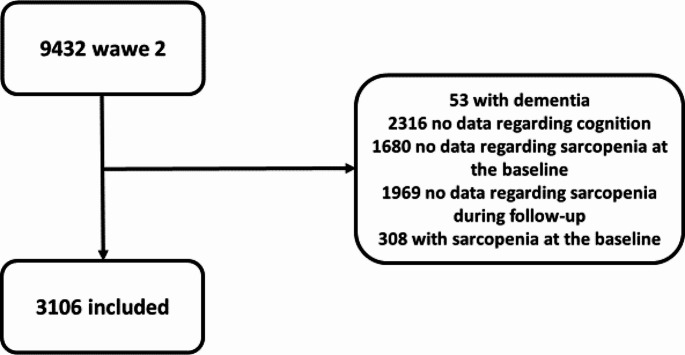
Flow chart

Figure 1 shows the flow chart of this research: among the 9,432 participants initially considered in the wave 2 of the ELSA study, we excluded 53 participants because they had dementia, 2,316 for whom no cognitive test data were available, 1,680 for whom no sarcopenia data were available, and 380 who already had sarcopenia at baseline. Moreover, 1,969 participants had insufficient information on incident sarcopenia, leaving 3,106 participants for this analysis. The 3,106 participants included had a mean age of 63.1 years (range: 52–88) and were predominantly male (55.3%).

Table 1 shows the baseline characteristics according to the presence or absence of MCI at wave 2. People affected by MCI (*n* = 729) were significantly older and more frequently females than their 2,377 counterparts without MCI. Moreover, people with MCI were less educated than those without MCI (*p* < 0.0001 for all the comparisons). No other significant differences emerged for general and demographic characteristics. Regarding medical conditions, participants with MCI did not differ compared to people without this condition, in terms of CVD, high blood pressure, and the other conditions examined, except for a higher prevalence of cataract (12.4% vs. 10.4%, *p* = 0.04) (Table 1). Finally, as shown in Table 1, people with MCI reported significantly lower mean handgrip strength values and SMI (*p* < 0.0001), as well as a higher prevalence of obesity (*p* = 0.001).

**Table 1:** Baseline characteristics by the presence or not of mild cognitive impairment

Parameter	No mild cognitive impairment (*n* = 2377)	Mild cognitive impairment (*n* = 729)	*p*-value
Demographics and general characteristics			
**Age, years**	62.7 (7.4)	64.4 (8.1)	< 0.0001
**Female sex**	42.4	52.1	< 0.0001
**Whites**	99.0	98.4	0.16
**Married**	95.8	96.4	0.43
**Years of education**	9.2 (6.5)	7.8 (6.8)	< 0.0001
**Ever smoking**	62.1	61.3	0.70
**High physical activity level**	24.6	26.6	0.56
***Medical conditions***			
**CES-D**	1.1 (1.6)	1.2 (1.7)	0.49
**Any CVD or diabetes **	25.9	24.2	0.15
**High blood pressure**	36.9	40.0	0.20
**Lung disease (including asthma)**	4.9	5.8	0.63
**Cancer**	7.4	5.6	0.29
**Parkinson’s disease**	0.5	0.2	0.79
**Psychiatric disorders**	9.6	7.9	0.41
**Glaucoma**	4.3	4.7	0.86
**Macular degeneration**	1.6	1.7	0.98
**Cataract**	10.4	12.4	0.04
***Sarcopenia parameters***			
**Mean handgrip strength**	33.7 (10.9)	31.6 (10.0)	< 0.0001
**SMI**	0.61 (0.18)	0.57 (0.18)	< 0.0001
**Obesity**	26.2	31.1	0.001

Abbreviations: CES-D: Center for Epidemiologic Studies Depression Scale, CVD: Cardiovascular Disease, SMI: Skeletal Mass Index

During the ten years of follow-up, 377 participants became sarcopenic, representing 12.1% of the initial population included. Table 2 shows the data of the multivariable logistic regression analysis using as outcome the incidence of sarcopenia. Among the factors considered, the presence of MCI (OR = 1.236; 95%CI: 1.090–1.596, *p* = 0.01) significantly predicted the onset of sarcopenia. Similarly, each year of increasing age increased the risk of sarcopenia by 6%, whilst not smoking was associated with a decreased risk in sarcopenia onset (OR = 0.517; 95%CI: 0.412–0.649; *p* < 0.0001). Among medical conditions investigated, only cataract was associated with a significantly higher risk of sarcopenia during follow-up by approximately 25% (Table 2). Finally, high physical activity was associated with a lower risk of sarcopenia during the follow-up period (OR = 0.716; 95%CI: 0.621–0.825; *p* < 0.0001), as well as an extra point in CES-D was associated with a 16% increased risk of sarcopenia (Table 2).

**Table 2:** Multivariate analysis of predictors of sarcopenia during follow-up

Parameter	Reference	Odds ratio	95% lower CI limit	95% higher CI limit	*p*-value
**MCI**	No MCI	1.236	1.090	1.596	0.01
**Age**	Increase in one year	1.066	1.050	1.082	< 0.0001
**Female sex**	Male sex	1.00	0.95	1.05	0.99
**Whites**	No whites	Too few cases			
**Married**	Other marital status	1.140	0.867	1.498	0.349
**Years of education**	Increase in one year	0.843	0.681	1.042	0.114
**No smokers**	Ever smokers (actual or previous)	0.517	0.412	0.649	< 0.0001
**High physical activity level**	Low physical activity, moderate phyiscal activity,sedentary	0.716	0.621	0.825	< 0.0001
**CES-D**	Increase in one point	1.162	1.088	1.241	< 0.0001
**Any CVD or diabetes**	No CVD or diabetes	0.889	0.634	1.245	0.493
**High blood pressure**	No high blood pressure	1.002	0.828	1.214	0.980
**Lung disease**	No lung disease	1.162	0.819	1.650	0.400
**Cancer**	No cancer	0.918	0.656	1.286	0.619
**Parkinson’s disease**	No Parkinson’s disease	Too few cases			
**Psychiatric disorders**	No psychiatric disorders	0.754	0.286	1.654	0.345
**Glaucoma**	No glaucoma	0.721	0.451	1.153	0.172
**Macular degeneration**	No macular degeneration	0.525	0.245	1.126	0.098
**Cataract**	No cataract	1.254	1.004	1.567	0.046
**Obesity**	Underweight, normal weight, overweight	1.715	0.280	10.444	0.342

Abbreviations: CES-D: Center for Epidemiologic Studies Depression Scale, CVD: Cardiovascular Diseases

## Discussion

To the best of our knowledge, this is one the first studies to assess the role of MCI as putative risk factor for sarcopenia. Our study, based on a large cohort of UK older adults, demonstrates that the presence of MCI at the baseline is significantly associated with a higher incidence of sarcopenia at 10-years of follow-up, showing a probable association between these two factors in older adults.

Our findings support that of previous studies. One study carried out in South-America worked on 521 community-dwelling older adults already identified MCI as predictor of sarcopenia, that is MCI at baseline predicted sarcopenia at 9-years of follow-up [40]. Another study in Japan including 250 older adults, also found a significant association between sarcopenia and MCI [41], however, it is important to note that this studies utilized two screening tools (SARC-F-J to assess sarcopenia and TYM-J to assess MCI).

Our study revealed how people affected by MCI were significantly older and more frequently females than their counterparts without MCI. Moreover, people with MCI were less educated than those without MCI. A recent study carried out in China on 1,325 participants aged ≥ 60 years found that the overall prevalence of MCI was higher in female compared to male participants, and female subjects who were illiterate had a higher risk of MCI, supporting findings from the present study [42]. . Regarding medical conditions, participants with MCI did not differ compared to people without this condition, except for a higher prevalence of cataract. Our results are supported by a previous review, that showed how visual impairment due to cataract may stress impaired attentional mechanisms, and cataract extraction may improve cognitive performance in some patients with MCI [42]. Similarly, we found that depression could be associated with sarcopenia: previous literature suggests that sarcopenia and depression often coexists [43], but it was poorly explored depression as potential risk factor for sarcopenia. Finally, among the factors explored potentially associated with sarcopenia, we found that higher physical activity level was protective: our finding is in agreement with the literature about this topic as also shown in a large systematic review about the topic [44]. 

MCI could predict sarcopenia through several mechanisms. First, sarcopenia leads to loss of muscle mass and muscle weakness and contributes to abnormal myokine secretion in the skeletal muscle, such as IL-6, IL-8, IL-15, and Brain-Derived Neurotrophic Factor (BDNF) [45]. These alterations of myokine levels in blood vessels during sarcopenia induce brain function in a paracrine and autocrine manner [46]. Myokine signaling may also explain the beneficial effects of physical activity on cognition in older adults, with an increase in the activity of prefrontal cortex and hippocampus, two brain regions involved in memory and cognition [47]. It is widely known that insulin resistance, oxidative stress, and low-grade chronic elevation of pro-inflammatory markers may be involved in both the pathogenesis of sarcopenia and cognitive impairment [48]. In fact, atherosclerosis is known as a process of chronic inflammation and alteration of the immune response, which leads to cardiovascular diseases [49]. Several studies have reported that patients with atherosclerosis showed a reduction in skeletal muscle function, skeletal muscle mass, and exercise intolerance, which are related to prevalence of sarcopenia [50].

Neurological factors also play a role in the pathophysiology of sarcopenia. Neuromuscular junctions (NMJs), while having the structural features of other chemical synapses, act as a bridge between the nervous (motor neuron) and skeletal muscle (myofiber) systems. NMJs play a relevant role in age-related musculoskeletal impairment [45]. Elevated serum levels of C-terminal agrin fragment (CAF), resulting from NMJ disassembly and denervation, are associated with sarcopenia [51], supporting the hypothesis that NMJs integrity is essential for the preservation of both motor nerve and muscle fibers [51]. 

Multidomain interventions may contribute to delaying the onset of sarcopenia in patients with MCI, with regard to physical, cognitive and dietary pattern. Engaging in regular physical activity, particularly aerobic exercise, has the potential to attenuate mitochondrial dysfunction and oxidative damage within motor neurons and NMJs [52]. . Aerobic exercise also appears to convey beneficial effects by maintaining an adequate release of neurotrophins that preserve the neuromuscular system [53]. Neuroimaging research has revealed a connection between cognitive abilities and gait control [45]. Cognitive therapy has been shown to be effective in preventing falls, and programs focused on walking have been found to lower the risk of dementia. Consequently, individuals experiencing cognitive decline demonstrate a slower walking pace than those in the control group [54]. Malnutrition is a risk factor for cognitive impairment, which is common among people with dementia, and associated with sarcopenia [55]. Numerous findings suggest that the adoption of healthy lifestyle habits serves to safeguard against cognitive decline [56]. The Mediterranean diet could also be a favourable option for older adults with sarcopenia to avoid or postpone cognitive decline [57].

Over ten years of follow-up, 12.1% of the initial population included in the present study became sarcopenic and the presence of MCI significantly predicted the onset of sarcopenia. Each year of increasing age increased the risk of sarcopenia by 6%, whereas not smoking was associated with a decreased risk in sarcopenia onset, as shown in a recent meta-analysis indicating that cigarette smoking as an isolated factor may contribute to the development of sarcopenia [58]. 

The results of this study must be interpreted considering its limitations. Firstly, the ELSA study did not include a multi-domain cognitive assessment, and functions such as psychomotor speed or executive function, which are often impaired in older subjects with cognitive decline, were not evaluated; this may have led to an underestimation of some cases of MCI, particularly those with attentional-executive impairment. Second, in ELSA, dementia diagnoses are not based on hospital data, but are based on the use of self-reported medical diagnoses, with the possibility of missing some cases with dementia at onset. Third, the ELSA study predominantly includes white British and thus results are not generalizable to over ethnicities. Fourth, body composition was not estimated using objective gold standard measurements: consequently, we had a surrogate of low fat-free mass, based on anthropometric parameters. Although no direct assessment of body composition was performed, the equation proposed by Lee and colleagues has good agreement with the gold standard tool for evaluating body composition, i.e. DXA [59]. Fifth, although the analyses were adjusted for the main potential confounders, residual confounding (e.g. neuropsychiatric symptoms, use of psychotropic drugs) cannot be excluded.

In conclusion, the data from the present work show that the presence of MCI at baseline was significantly associated with a higher incidence of sarcopenia at 10-years of follow-up, demonstrating a probable role of MCI as a predictor of the onset of sarcopenia among older persons, highlighting how the nervous and muscular systems are closely related, as already expressed for millennia by the Latin proverb *mens sana in corpore sano*. These findings suggest that interventions are needed to help prevent sarcopenia among older people with MCI, thereby reducing the burden on individuals, families and society and, subsequently, increasing quality of life. The data of the present study need to be confirmed by prospective population studies conducted on subjects with MCI defined by means of multidimensional cognitive batteries.

## Data Availability

No datasets were generated or analysed during the current study.

## Declarations

### Data sharing

The study protocol and statistical analysis plan for this project are available on request from the corresponding author. Data are available from the UK Data Service for researchers who meet the criteria for access to confidential data. Data are from waves 2 to 8 of the ELSA study. Data and contact details may be obtained via the website http://www.adls.ac.uk/find-administrative-data/linked-administrative-data/english-longitudinal-study-of-ageing/
